# Emphysema-related mortality rates in the U.S. from 1999 to 2022

**DOI:** 10.3389/fmed.2025.1579177

**Published:** 2025-05-14

**Authors:** Alexandra Brown, Amanda Karl, Vikram Murugan, Taylor Billion, Ali Bin Abdul Jabbar, Mohsin Mirza

**Affiliations:** ^1^School of Medicine, Creighton University, Omaha, NE, United States; ^2^Department of Internal Medicine, Creighton University, Omaha, NE, United States

**Keywords:** CDC WONDER, CDC WONDER database, emphysema, COPD, age-adjusted mortality rate

## Abstract

**Introduction:**

Pulmonary emphysema is a progressive lung disease characterized by persistent respiratory symptoms that are a result of destruction to the alveoli wall and enlargement of distal airspaces. Despite initiatives made to create awareness about the dangers of smoking, and a nationwide reduction in cigarette smoking, emphysema (COPD) is still the third leading cause of death in the United States.

**Methods:**

This study utilized the CDC WONDER national database to investigate the trends in emphysema-related mortality in the United States. Age-adjusted mortality rates per 100,000 people (AAMR), annual percentage change (APC), and average annual percentage change (AAPC) with 95% confidence intervals (CIs) were assessed. The Joinpoint Regression Program was used to determine mortality trends between 1999 and 2022. Data extracted for analysis in this study includes gender, race/ethnicity, age groups, regions, states, and urban/rural classification.

**Results:**

From 1999 to 2022, there were 526,545 deaths due to emphysema in the United States. Overall age-adjusted mortality rates (AAMR) in the United States decreased from 18.47 in 1999 to 7.75 in 2022, with an average annual percentage change (AAPC) of −3.698. Emphysema caused 296,859 deaths in males and 229,686 in females in the United States. White populations had the highest AAMR over this period and the largest reduction in AAMR. AAMRs were initially highest in urban areas compared to rural regions. 85 + years had the highest crude mortality rate of 123.11 in 1999.

**Discussion:**

Emphysema-related deaths in the United States decreased overall between 1999 and 2022, likely a result of a greater emphasis on health education concerning the significant dangers of smoking and policy changes that made cigarettes less accessible and less affordable, and more available access to resources and support networks.

**Conclusion:**

It is important to address possible health disparities that exist among populations and improve healthcare outcomes and resource allocation among all population groups.

## Introduction

Pulmonary emphysema is considered a sub-type of Chronic Obstructive Lung Disease (COPD). It is a progressive lung disease characterized by persistent respiratory symptoms resulting from destruction to the alveoli wall and permanent enlargement of distal airspaces (1). It has been well-documented that emphysema is frequently the result of chronic exposure to toxins, most notably cigarette smoke as it remains the most common cause of emphysema in the United States. As well as cigarette smoke, exposure to other toxic substances have shown to play a role in disease development, including asbestos, workplace exposure to chemical fumes (including silica dust, welding fumes, coal dust etc.) and certain pesticides and herbicides (2). In response to persistent exposure to cigarette smoke, inflammatory cells are recruited resulting in downstream effects including proteinase secretion, mucus hypersecretion, and large cytokine release, that damage the alveoli and distal airways (3). Life expectancy varies with the severity of the disease, with the mean life expectancy for milder cases being approximately ten years (4).

Despite initiatives made to create awareness about the dangers of smoking and a nationwide reduction in cigarette smoking, it is worth noting that emphysema (COPD) is still the third leading cause of death in the United States and the fourth leading cause of death worldwide (3). Emphysema often coexists with various comorbidities that have been shown to increase mortality and morbidity in those living with the disease, including cachexia and muscle wasting, and osteoporosis (1). Current pharmacological therapies available for patients with emphysema have shown improved lung function and clinical outcomes (5). The current disease management strategies include bronchodilators, systemic steroids, antibiotics, and oxygen therapy (5). Despite the development of various pharmacotherapies to help manage this disease, costs to the U.S. healthcare system continue to increase with therapeutic outpatient management and hospitalizations from acute exacerbations (6). Additionally, while medical management can slow disease progression and help prevent frequent hospitalizations, there is, at present, no cure for emphysema, and patients continue to deteriorate despite the variety of medication options available to them (5). For this reason, prevention strategies—including education surrounding the dangers of smoking and the importance of avoiding lung irritants (such as second-hand smoke, chemical toxins, air pollutants, etc.), as well as the benefits of exercise—are all extremely important in decreasing the emphysema disease burden in the United States.

This study utilized the Centers for Disease Control and Prevention Wide-Ranging Online Data for Epidemiologic Research (CDC WONDER) national database to investigate the trends in emphysema-related mortality in the United States. CDC WONDER provides a wide range of public health data including births, deaths, cancer diagnoses, vaccinations, and environmental exposures (7). Other databases such as Surveillance, Epidemiology, and End Results (SEER), which is managed by the National Cancer Institute (NCI), utilizes population-based cancer registries across the United States, and is considered the most reliable source for cancer incidence and survival statistics in the U.S. (8). The National Cancer Database (NCDB) is another clinical oncology database sourced from more than 1,500 Commission on Cancer-accredited facilities to track patient treatments and outcomes (9). The SEER and NCDB databases, which are focused on cancer diagnoses, would be less useful for our objective of investigating emphysema deaths specifically.

Previous research has utilized CDC WONDER between 2004 and 2018 to demonstrate that COPD mortality has decreased among Americans (10). However, upon closer inspection, it is clear that a dramatic decrease in mortality rates was not observed among all demographic groups within the United States (10). This study will expand on past research by looking at emphysema deaths in a wider time frame and in more recent years (1999 to 2022). Data extracted for analysis in this study includes gender, race/ethnicity, age groups, region, state, and urban/ rural classification. Additionally, while past literature broadly examines COPD, this study is focused on emphysema-related mortality, a subtype of COPD.

## Methods

Centers for Disease Control and Prevention Wide-ranging Online Data for Epidemiologic Research (CDC WONDER) was used to identify emphysema-related deaths in the United States (11). This database has been previously used in several other studies to analyze nationwide trends in mortality of COPD (12) but with no emphasis on emphysema specifically. The methodology employed with this study is very similar to previous studies that have been conducted, but with the focus on the Eighth Revision, International Classification of Diseases, Adapted for Use in the United States (ICD-8), code J43 – emphysema-related deaths in patients ≥25 years from 1999 to 2022. This age restriction was selected because emphysema is infrequent in patients <25 years, and we wanted to focus on the older population due to the high prevalence of this disease that is seen within this demographic. The study was exempt from institutional review board approval because the CDC WONDER database contains anonymized, publicly available data.

As seen in similar studies, the data on demographic and regional groups were extracted, including gender, race/ethnicity, age, urban–rural classification, region, and states. Specifically, age groups were defined as 25–34, 35–44, 45–54, 55–64, 65–74, 75–84, and 85 + years of age. Emphysema-related crude and age-adjusted mortality rates were calculated. Crude mortality rates were calculated by dividing the number of AMI-related deaths by the corresponding United States population. AAMR was standardized using the 2000 United States standard population as previously described (13). The Joinpoint Regression Program (Joinpoint version 4.9.0.0 available from National Cancer Institute, Bethesda, Maryland) was used to determine trends in mortality within the study period (14). Annual percentage change (APC) with 95% confidence intervals (CIs) for the AAMRs were calculated for the line segments linking a Joinpoint using the Monte Carlo permutation test. The weighted average of the APCs was calculated and reported as AAPCs and corresponding 95% CIs to summarize the reported mortality trend for the entire study period. APC and AAPCs were considered increasing or decreasing if the slope describing the change in mortality over the time interval was significantly different from zero using a 2-tailed *t*-test. Statistical significance was set at *p* ≤ 0.05.

## Results

### Overall

From 1999 to 2022, there were 526,545 deaths due to emphysema in the United States. Overall age-adjusted mortality rates (AAMR) in the United States decreased during the period of 1999 to 2022 from 18.47 in 1999 to 7.75 in 2022, with an average annual percentage change (AAPC) of −3.698. The annual percentage change (APC) in AAMR was statistically significant at −8.922 from 1999 to 2001, which decreased to −3.796 and was also statistically significant from 2001 to 2008. It was −8.377 from 2008 to 2015 and a statistically significant value of 2.951 from 2015 to 2022. Overall, AAMR decreased to its lowest point in 2020 and increased from 7.24 in 2020 back up to 7.75 in 2022 (Supplementary Figure 1; Supplementary Table 1).

### Gender

From 1999 to 2022, emphysema caused 296,859 (0.0125%) deaths in males and 229,686 (0.00899%) in females in the United States.

**Figure 1:**
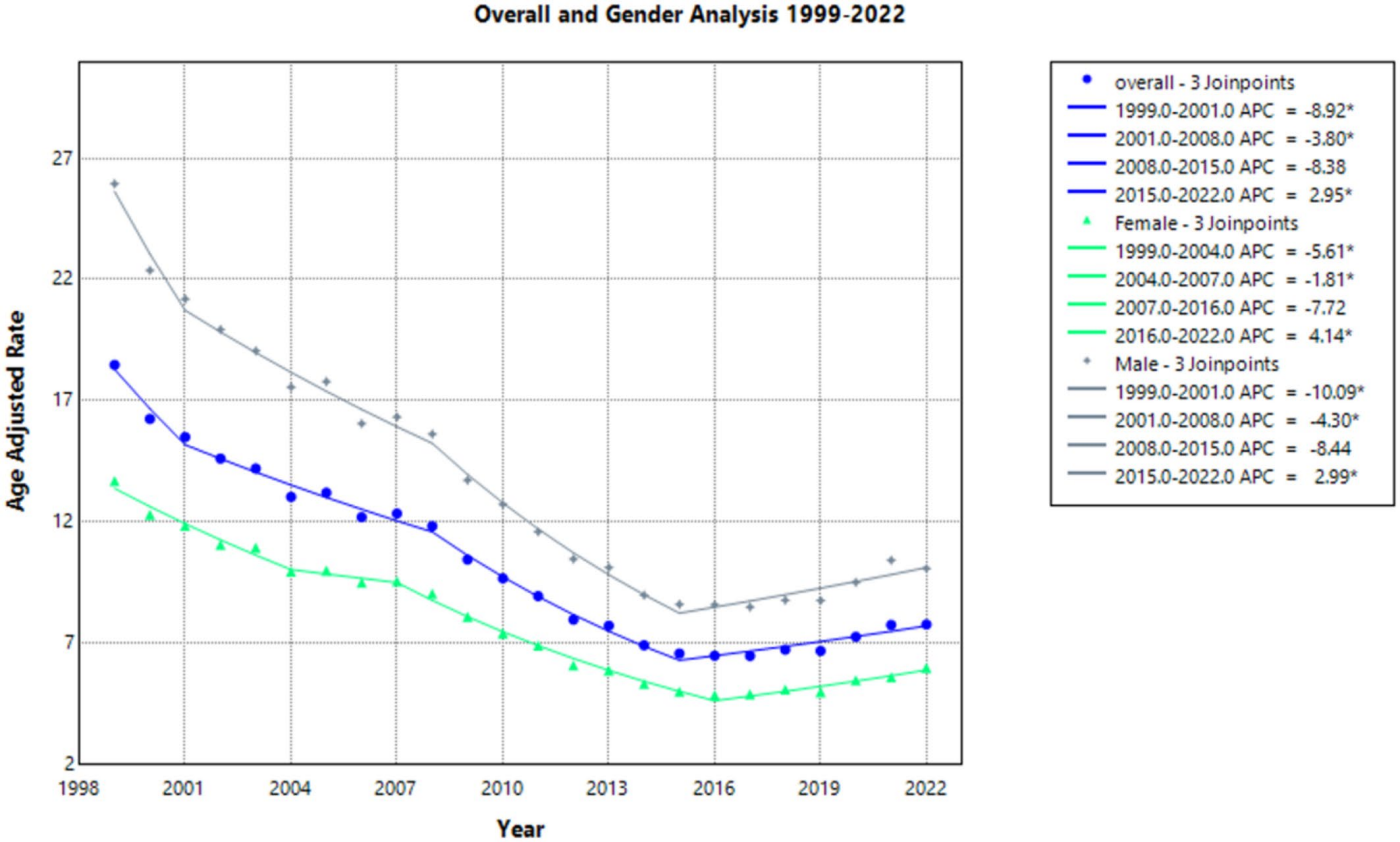
Overall and gender joinpoint analysis of emphysema AAMR per 100,000 residents, 1999–2022.

The AAMR decreased in females from 13.67 in 1999 to 5.96 in 2022 (Supplementary Figure 1; Supplementary Table 1), with a statistically significant AAPC of −3.518. As seen in Figure 1, the APC in AAMR was statistically significant at −5.614 from 1999 to 2004, which then decelerated to a statistically significant value of −1.809 from 2004 to 2007. From 2007 to 2016, it was −7.723. It was then a statistically significant value of 4.140 from 2016 to 2022.

In males, the AAMR decreased from 25.95 in 1999 to 10.06 in 2022 (Supplementary Figure 1; Supplementary Table 1), with a steady statistically significant AAPC of −3.968. The APC in AAMR was statistically significant at −10.087 from 1999 to 2001. From 2001 to 2008, it was a statistically significant value of −4.302. It was −8.437 from 2008 to 2015 and a statistically significant value of 2.990 from 2015 to 2022 (Figure 1).

### Race

**Figure 2:**
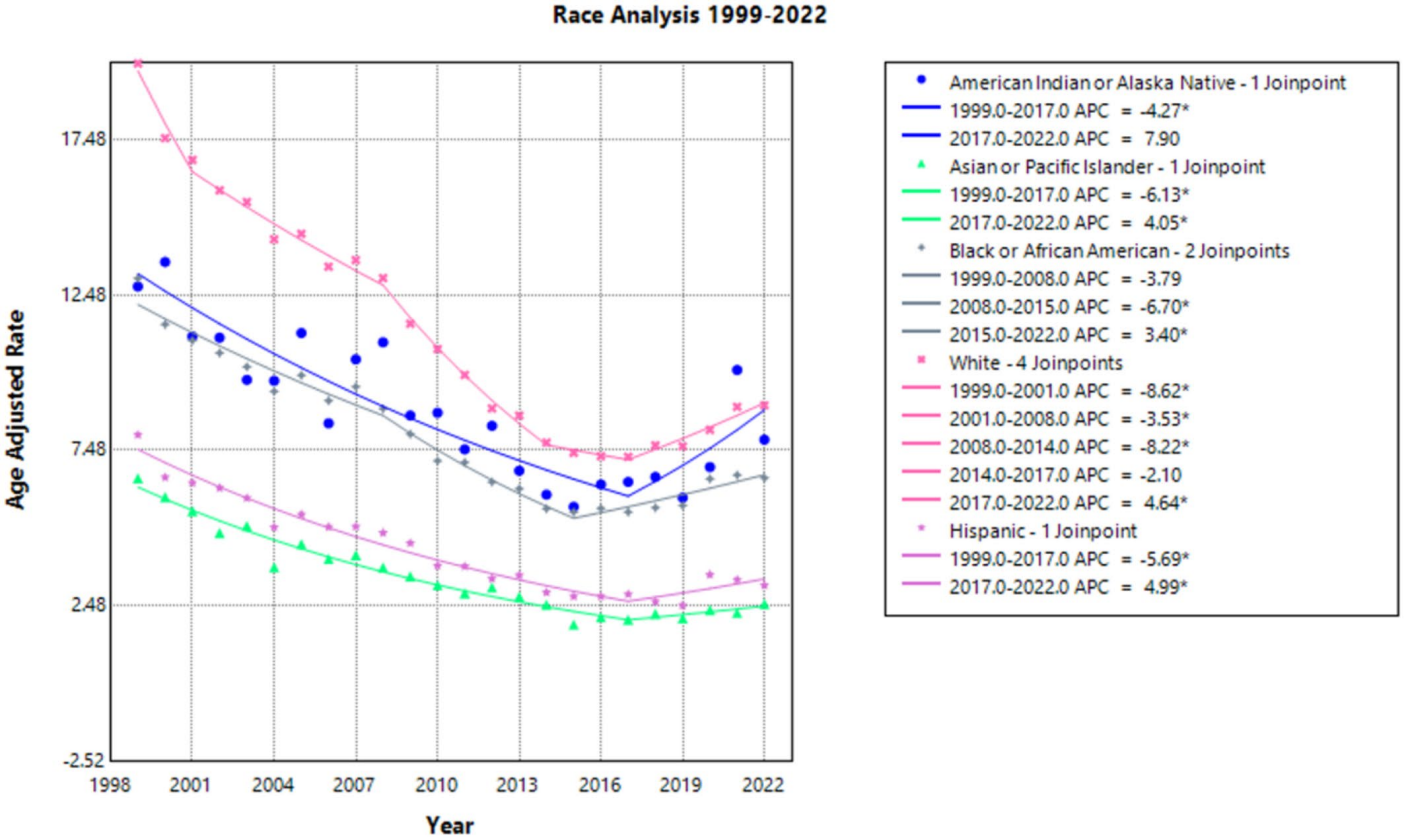
Race joinpoint analysis of emphysema AAMR per 100,000 residents, 1999–2022.

White people had the highest AAMR over the years (and the largest reduction in AAMR), with 19.95 in 1999 to 8.93 in 2022 (Supplementary Figure 2; Supplementary Table 2) and a statistically significant AAPC of −3.348. The APC was −8.619 from 1999 to 2001, −3.526 from 2001 to 2008, and −8.223 from 2008 to 2014—all of which were statistically significant. It reduced to −2.098 from 2014 to 2017. Finally, it was a statistically significant value of 4.638 from 2017 to 2022 (Figure 2).

Asian or Pacific Islander people had the lowest AAMR consistently from 6.58 in 1999 to 2.53 in 2022 (Supplementary Figure 2; Supplementary Table 2) with an AAPC of −4.005, which was statistically significant. The APC was −6.131 from 1999 to 2017 and then 4.054 from 2017 to 2022, both of these values were statistically significant (Figure 2).

The American Indian and Native Alaskan had an AAMR of 12.76 in 1999 and 7.82 in 2022 (Supplementary Figure 2; Supplementary Table 2). The AAPC between 1999 and 2022 was statistically significant at −1.747. APC accelerated at a statistically significant rate of −4.270 from 1999 to 2017 and subsequently increased to 7.897 from 2017 to 2022 (Figure 2).

Black or African American population AAMR decreased from 13.02 in 1999 to 6.59 in 2022 (Supplementary Figure 2; Supplementary Table 2), with a statistically significant AAPC of −2.571. The APC in AAMR was −3.791 from 1999 to 2008. It subsequently was −6.700 from 2008 to 2015 and then increased to 3.404 from 2015 to 2022, both of which were statistically significant (Figure 2).

Finally, the Hispanic population AAMR decreased from 7.98 in 1999 to 3.13 in 2022 (Supplementary Figure 2; Supplementary Table 2). The AAPC was statistically significant at −3.467 between 1999 and 2022. The APC was −5.692 from 1999 to 2017 and subsequently was 4.986 from 2017 to 2022, both statistically significant values (Figure 2).

### Rural/urban regions

**Figure 3:**
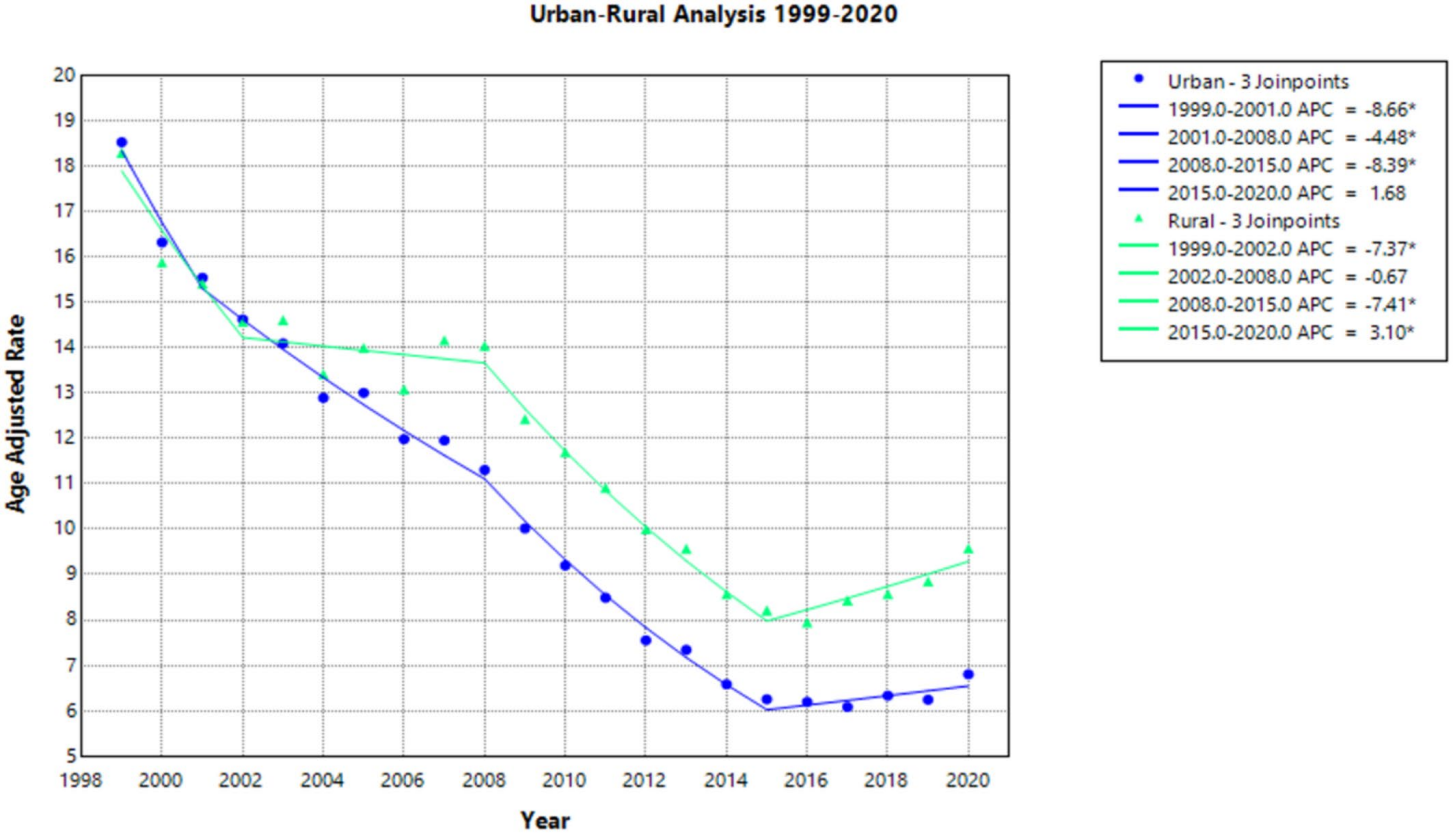
Urban–rural joinpoint analysis of emphysema AAMR per 100,000 residents, analysis 1999–2020.

When comparing populated regions, AAMRs were initially highest in urban areas compared to rural regions (Supplementary Figure 3; Supplementary Table 3). Urban zones saw AAMRs decline from 18.54 in 1999 to 6.80 in 2020, with a consistent statistically significant AAPC of −4.793. The APC in AAMR was −8.664 from 1999 to 2001, −4.476 from 2001 to 2008, and −8.386 from 2008 to 2015—all of these APCs were statistically significant. The APC in AAMR from 2015 to 2020 was then 1.685, which was not statistically significant (Figure 3).

Rural areas also experienced a decline in AAMR, but less so than in urban areas (Supplementary Figure 3; Supplementary Table 3). The AAMR for rural regions decreased from 18.31 in 1999 to 9.58 in 2020, with a consistent statistically significant AAPC of −3.076. For the period of 1999–2002, APC in AAMR was a statistically significant value of −7.369. For the period of 2002–2008, the APC was found to be −0.668. For the period of 2008–2015, the APC was found to be −7.406, which was statistically significant. For the period of 2015–2020, the APC was found to be 3.096, which was also statistically significant (Figure 3).

### Regions

**Figure 4:**
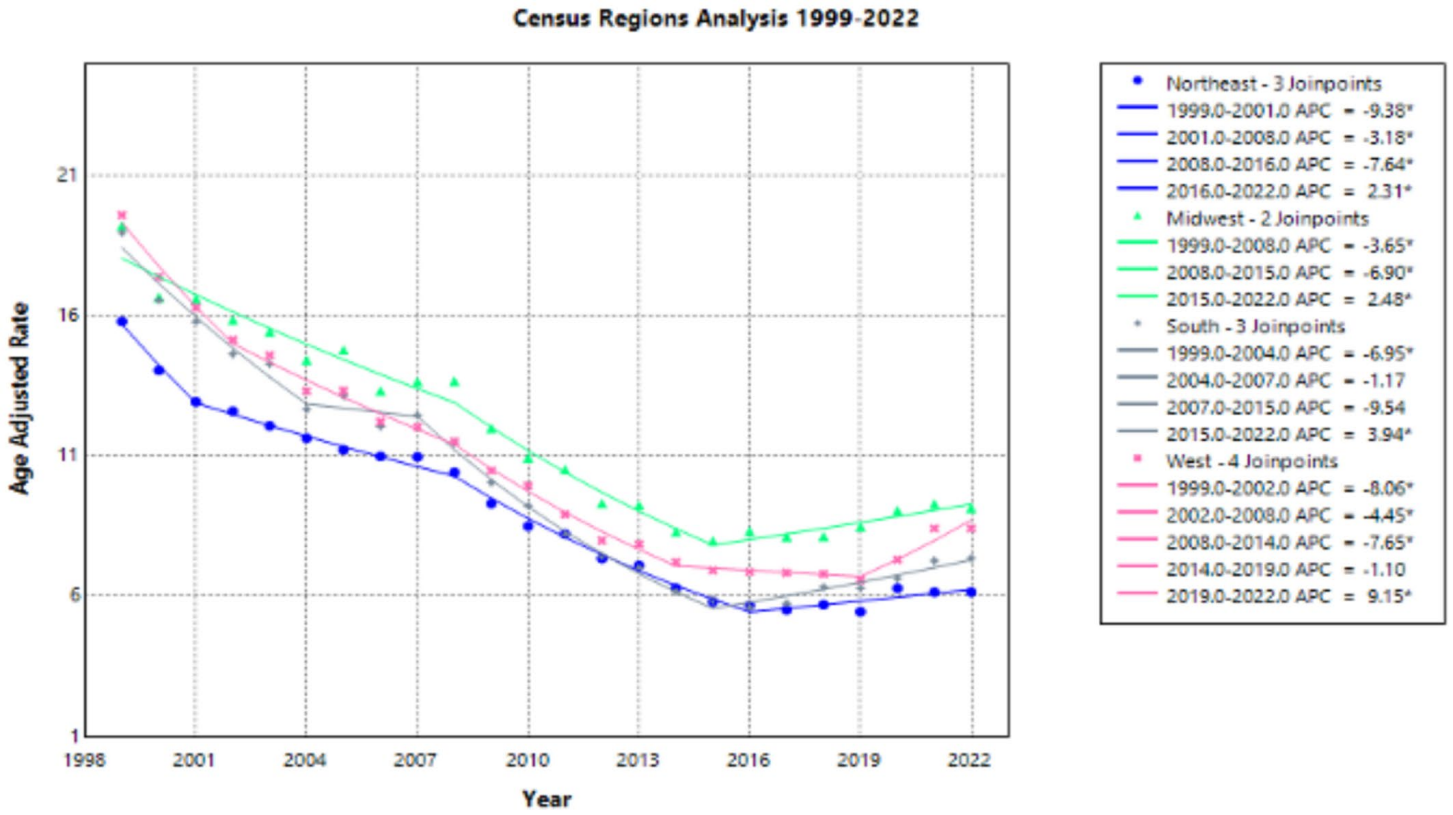
Census regions joinpoint analysis of emphysema AAMR per 100,000 residents, 1999–2022.

All census regions showed an overall decrease in AAMR from 1999 to 2022 (Supplementary Figure 4; Supplementary Table 4). In 1999, the West displayed the highest AAMR of 19.59, with a decrease to 8.42 in 2022. The AAPC for the West showed a significant value of −3.400 for the interval 1999–2022. The APC in AAMR had a statistically significant value of −8.060 from 1999 to 2002, a statistically significant value of −4.455 from 2002 to 2008, a statistically significant value of −7.653 from 2008 to 2014, a value of −1.097 from 2014 to 2019, and a statistically significant value of 9.153 from 2019 to 2022 (Figure 4).

Data from the Northeast revealed an AAMR of 15.8 in 1999, with a decrease to 6.15 in 2022 (Supplementary Figure 4; Supplementary Table 4). The AAPC for the Northeast from 1999 to 2022 showed a statistically significant value of −3.931. The APC in AAMR had a statistically significant value of −9.380 from 1999 to 2001, a statistically significant value of −3.179 from 2001 to 2008, a statistically significant value of −7.644 from 2008 to 2016, and a statistically significant value of 2.310 from 2016 to 2022 (Figure 4).

The Midwest displayed an AAMR of 19.22 in 1999, with a decrease to 9.15 in 2022 (Supplementary Figure 4; Supplementary Table 4). The AAPC for the Midwest from 1999 to 2022 showed a statistically significant value of −2.846. The APC in AAMR had a statistically significant value of −3.653 from 1999 to 2008, a statistically significant value of −6.900 from 2008 to 2015, and a statistically significant value of 2.476 from 2015 to 2022 (Figure 4).

Finally, data from the South depicted an AAMR of 18.97 in 1999, with a decrease to 7.36 in 2022 (Supplementary Figure 4; Supplementary Table 4). The AAPC for the South from 1999 to 2022 showed the greatest statistically significant value for each of our four regional variables of −3.947. The APC in AAMR had a statistically significant value of −6.948 from 1999 to 2004, a value of −1.165 from 2004 to 2007, a value of −9.537 from 2007 to 2015, and a value of 3.943 from 2015 to 2022 (Figure 4).

### States

For the interval 1999–2019, the state with the largest decrease in AAMR was Michigan with a crude mortality rate of −18.14 (Supplementary Figure 5). Georgia saw the second-largest decrease with an AAMR of −16.86. Texas saw the third-largest decrease with an AAMR of −16.32. For this same time period, North Dakota had the smallest change in AAMR with a value of −2.09, followed by Minnesota with an AAMR of −2.24, then Rhode Island with an AAMR of −2.72. For the interval 2019–2022, the state with the largest positive AAMR was New Mexico with a value of 6.15, followed by Oklahoma with an AAMR of 5.79, followed by West Virginia with an AAMR of 5.6 (Supplementary Figure 6). For this same time period, Tennessee had the largest negative AAMR with a value of −6.68, followed by Montana with an AAMR value of −3.59, and lastly followed by Kentucky with an AAMR value of −1.65.

### Age

**Figure 5:**
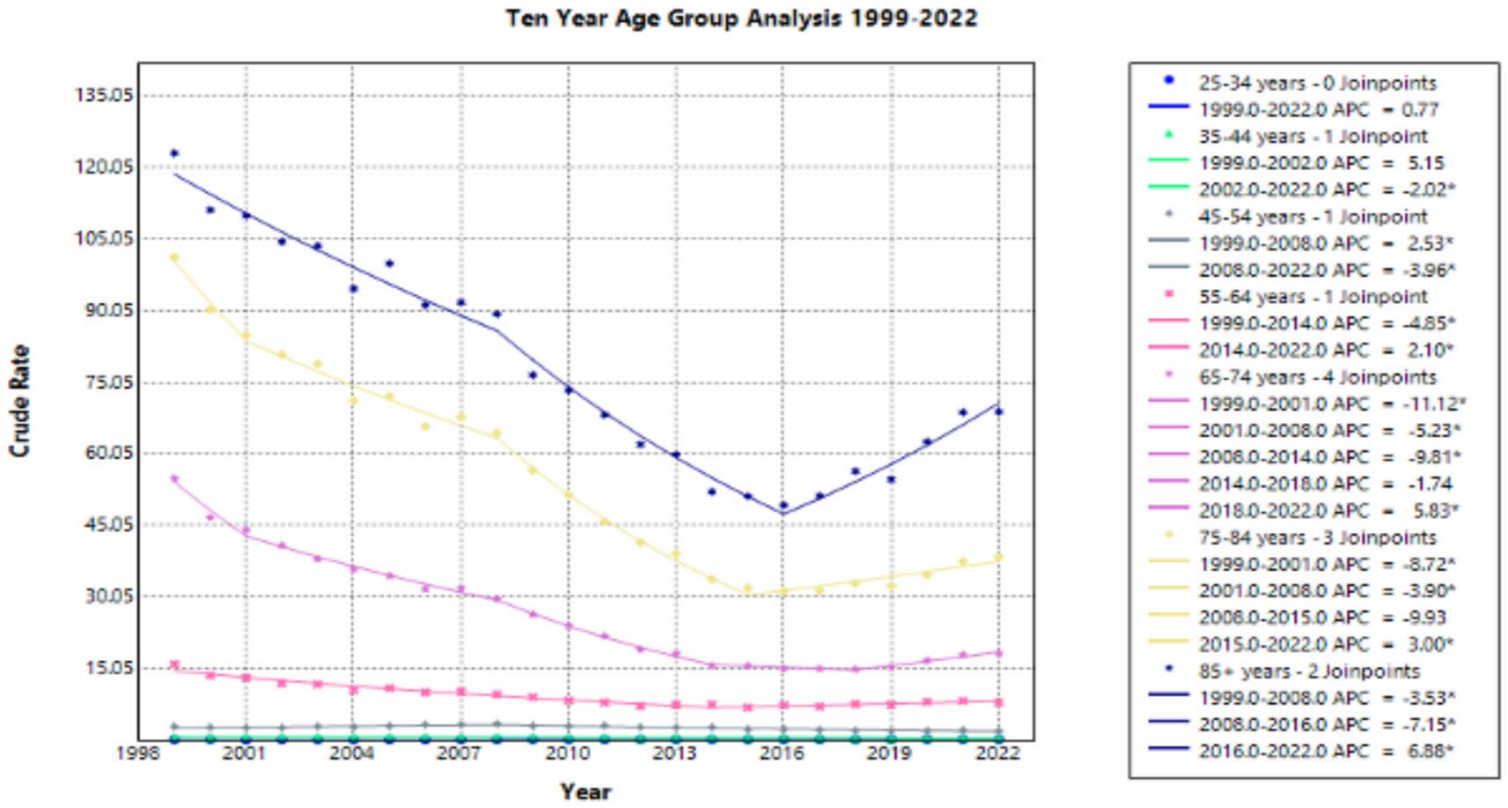
Ten year age group joinpoint analysis of emphysema crude rate mortality per 100,000 residents, 1999–2022.

For the age categories, 85 + years had the highest crude mortality rate of 123.11 in 1999 (Supplementary Figure 7; Supplementary Table 5). This crude rate dropped to 68.83 in 2022. The AAPC for this age group from 1999 to 2022 was a statistically significant value of −2.228. The APC in crude mortality rate had a statistically significant value of −3.534 from 1999 to 2008, a statistically significant value of −7.154 from 2008 to 2016, and a statistically significant value of 6.883 from 2016 to 2022 (Figure 5).

For the age category 75–84 years, the crude mortality rate was 101.22 in 1999 and decreased to 38.41 in 2022 (Supplementary Figure 7; Supplementary Table 5). The AAPC for this age group from 1999 to 2022 was a statistically significant value of −4.199. The APC in crude mortality rate had a statistically significant value of −8.725 from 1999 to 2001, a statistically significant value of −3.904 from 2001 to 2008, a value of −9.933 from 2008 to 2015, and a statistically significant value of 3.003 from 2015 to 2022 (Figure 5).

For the age category 65–74 years, the crude mortality rate was 54.88 in 1999 and decreased to 18.19 in 2022 (Supplementary Figure 7; Supplementary Table 5). The AAPC for this age group from 1999 to 2022 was a statistically significant value of −4.565. The AAPC for this age group was a statistically significant value of 11.118 from 1999 to 2001, a statistically significant value of −5.226 from 2001 to 2008, a statistically significant value of −9.813 from 2008 to 2014, a value of −1.744 from 2014 to 2018, and a statistically significant value of 5.835 from 2018 to 2022 (Figure 5).

For the age category 55–64 years, the crude mortality rate was 15.93 in 1999 and decreased to 7.88 in 2022 (Supplementary Figure 7; Supplementary Table 5). The AAPC for this age group from 1999 to 2022 was a statistically significant value of −2.489. The APC in crude mortality rate had a statistically significant value of −4.852 from 1999 to 2014, and a statistically significant value of 2.102 from 2014 to 2022 (Figure 5).

For the age category 45–54 years, the crude mortality rate was 2.86 in 1999 and decreased to 1.9 in 2022 (Supplementary Figure 7; Supplementary Table 5). The AAPC for this age group from 1999 to 2022 was a statistically significant value of −1.473. The APC in crude mortality rate had a statistically significant value of 2.532 from 1999 to 2008, and a statistically significant value of −3.965 from 2008 to 2022 (Figure 5).

For the age category 35–44 years, the crude mortality rate was 0.55 in 1999 and decreased to 0.44 in 2022 (Supplementary Figure 7; Supplementary Table 5). The AAPC for this age group from 1999 to 2022 was a statistically significant value of −1.116. The APC in crude mortality rate had a value of 5.148 from 1999 to 2002, and a statistically significant value of −2.023 from 2002 to 2022 (Figure 5).

The age category 25–34 years had the lowest crude mortality rate of 0.07 in 1999 and this value remained the same for 2022 (Supplementary Figure 7; Supplementary Table 5). The AAPC for this age group from 1999 to 2022 was 0.773. The APC in crude mortality rate had a value of 0.773 from 1999 to 2022 (Figure 5).

## Discussion

The overall decrease in emphysema-related mortality between 1999 and 2022 is most likely linked to the nationally increasing emphasis on the dangers of smoking and policy changes that have made cigarettes both less accessible and more expensive. Emphysema is frequently the result of chronic exposure to toxins, most notably cigarette smoke. Persistent exposure to cigarette smoke causes damage to alveoli and distal airways, eventually causing emphysema, a subtype of chronic obstructive pulmonary disease (COPD) (3). Recent anti-smoking campaigns have focused on spreading information about the dangerous outcomes of smoking (15). These have been implemented in public education in the form of the FDA’s award-winning public education campaign, “The Real Cost” which discusses the dangers of nicotine used in the form of vape, cigarettes, and dip (16). Additional efforts are made in regard to advertisements on television for older demographics, or those who are already smoking and considering quitting. For example, the CDC’s campaign “Tips from Former Smokers” describes complications such as cancer, heart failure, and COPD which can come as a result of tobacco use (17). The *Tips* campaign also aims to “address health disparities in pursuit of health equity by increasing the reach, representation, receptivity, and accessibility of smoking cessation messages” with the ultimate goal of increasing awareness about resources for quitting that are available to anyone, regardless of income or zip code (17). One such resource available is called “Every Try Counts,” which provides quitting support, advice, and free online counseling for smokers at any stage of their journey.

In 2021, an estimated 11.5% (28.3 million) of U.S. adults smoked cigarettes. This explains why emphysema remains a leading cause of death despite the significant decrease in overall age-adjusted mortality rates (AAMR). “Current cigarette smoking” is defined as smoking ≥100 cigarettes during a lifetime and now smoking cigarettes either every day or some days (18). The AAMR was higher in males than in females during the entirety of this study’s time frame, from 1999 until 2022. At the time of the CDC’s 2021 survey, 13.1% of males over the age of 18 reported cigarette use “every day” or “some days,” while only 10.1% of females reported use (18). The consistently higher use of cigarettes in men may be due to differences in smoking dependence motives. For instance, studies have shown that women are more likely to continue smoking due to cue exposure, stress, or weight loss goals, while men often smoke in order to maintain nicotine levels (19). Additionally, AAMR increased with age, which is unsurprising given that cumulative damage from smoking can take many years to develop and the increasing prevalence of comorbidities that also arise with increasing age which may have played a significant role in the mortality rates observed (20).

There are also significant differences in emphysema-related mortality between racial groups. White individuals consistently had the highest AAMRs, while Asian or Pacific Islander people had the lowest AAMRs. The other studied groups that had intermediate mortality rates were Black or African American people, American Indian and Native Alaskan people, and finally the Hispanic population. As of the CDC’s survey in 2021, White people had the highest cigarette use among these racial groups, with 12.9% reporting smoking either “every day” or “some days,” followed by 11.7% of Black-identifying individuals reported smoking, 7.7% of Hispanic people, and 5.4% of Asian individuals. 2021 tobacco product estimates for American Indian/Alaska Native adults were not statistically reliable (17, 18). The variance in cigarette use between racial groups has been speculated to be a result of genetic ancestry and traits linked with smoking initiation or cessation, as well as different social and cultural norms (21). While the variation in genetic ancestry between Asian and Hispanic/Latino individuals and other populations may explain the differences in cigarette smoking in these groups, no study to date has investigated these specific genetic variances. However, people of lower socioeconomic status appear to have consistently higher rates of cigarette use (21). This could explain some of the racial group variations, as both structural and societal factors (and also zip code), when taken into account, often demonstrate the lower overall household income of many Black and Hispanic individuals in the United States.

Urban areas had higher age-adjusted mortality rates for emphysema than rural areas in 1999. However, there was a larger decrease in emphysema-related mortality between 1999 and 2020 for urban areas, leading to lower mortality in urban than rural populations in 2020. Findings from the 2010–2020 US National Survey on Drug Use and Health (NSDUH) demonstrated higher smoking prevalence and lower quit ratios in rural versus urban areas after adjusting for sociodemographic characteristics (22). This could explain, in part, the higher emphysema-related mortality in rural areas than in urban areas in recent years. These results may be caused by reports of higher nicotine dependence and heaviness of smoking among rural vs. urban residents. Rural residents may also face structural barriers to smoking cessation services, including lower rates of insurance coverage and fewer available healthcare providers (22). It is also worth mentioning that those living in urban areas are exposed to more air pollutants than those in rural areas, as this may also be a contributing factor to the observed results of this study. In terms of the discrepancies in data that we observed among states in the U.S., Texas had one of the highest decreases in AAMR. This could possibly be attributed to the Chronic Respiratory Disease Strategy Plan that was implemented by the Department of State Health Services in 2017, which sought to reduce chronic respiratory disease in the state of Texas (23).

Overall, emphysema-related mortality increased between 2020 and 2022, after it had been declining since 1999, with no concurrent rise in smoking rates, as might have been expected. This change could be explained by the COVID-19 pandemic occurring during this time period. Pre-existing COPD has been found to lead to worse COVID-19 outcomes. However, the exact relationship is difficult to elucidate due to confounding factors such as cigarette smoking, inhaled corticosteroid exposure, socioeconomic status, and genetics that may influence this association (24). Additionally, around 50% of COPD exacerbations are caused by respiratory viral infections. Therefore, a higher population prevalence of any viral infection (in this case, SARS-CoV-2) would likely cause more frequent exacerbations and hospitalizations for individuals with COPD (25). These associations between COPD and COVID-19 can help explain why age-adjusted mortality rates for emphysema increased across all analyzed variables in the years following the start of the COVID-19 pandemic.

It is worth noting that these findings represent extrapolations of mortality data, warranting further research and a better understanding of a patient’s comorbidities or risk factors for reliable conclusions to be made from this data. Limitations of the analysis discussed in this paper include a lack of individual-level risk factors or other possible comorbidities that may have contributed as important confounding variables for the results that were observed. Because of the limitations associated with the use of the CDC-WONDER database, information relating to family history, treatments, and past medical history has not been included in this analysis and may have contributed to the trends that were noted above.

It is therefore important to emphasize that causal conclusions cannot be reliably drawn from any of the results discussed above. Further research could benefit from the incorporation of additional factors that might have a notable influence on emphysema-related AAMR, such as other comorbidities including cardiac disease, diabetes mellitus, hypertension, osteoporosis, and psychological disorders, which are all commonly reported in patients with COPD. Addressing these factors alongside emphysema treatment could help improve the understanding of the underlying causes (26). To work towards increasing health equity throughout the United States, it is imperative to consider these differences and allocate resources effectively, in order to significantly minimize these differences in mortality trends and improve healthcare outcomes among all populations.

## Conclusion

The emphysema-related mortality rates among individuals across the U.S. from 1999 to 2022 have shown a significant decrease across age, race, gender, states, regions, and urbanization differences. A notable rise in AAMR was observed from 2020 to 2022, which could be attributed to the increased mortality rates that were observed during the COVID-19 pandemic. These findings underscore the importance of understanding the differences that are observed among specific racial populations and those living in certain regions of the United States who may be exposed to different cultural and societal norms, as well as subjected to various toxins and pollution. In conjunction with the current treatments that are available for emphysema, it is important to address the other possible health disparities that exist among populations in the U.S. in order to be able to best treat this disease.

## Data Availability

The raw data supporting the conclusions of this article will be made available by the authors, without undue reservation.
