# Supplementary material for: Emphysema-related mortality rates in the U.S. from 1999 to 2022
Source: Front Med (Lausanne). 2025 May 14;12:1579177. doi: 10.3389/fmed.2025.1579177 (PMC12116317; doi:10.3389/fmed.2025.1579177)
Supplement: Supplementary file 1 [file Image_1.pdf]

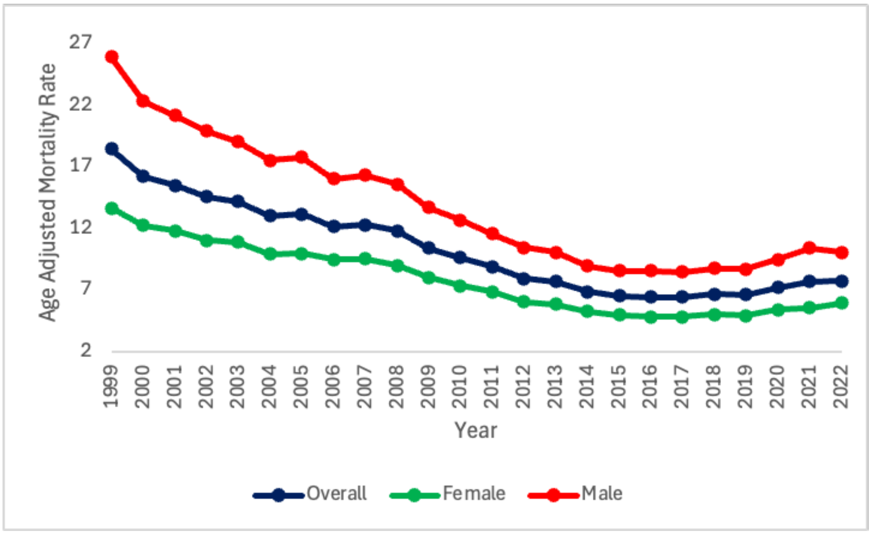

Supplemental Figure 1 - Emphysema Overall and Gender Age Adjusted Mortality Rates

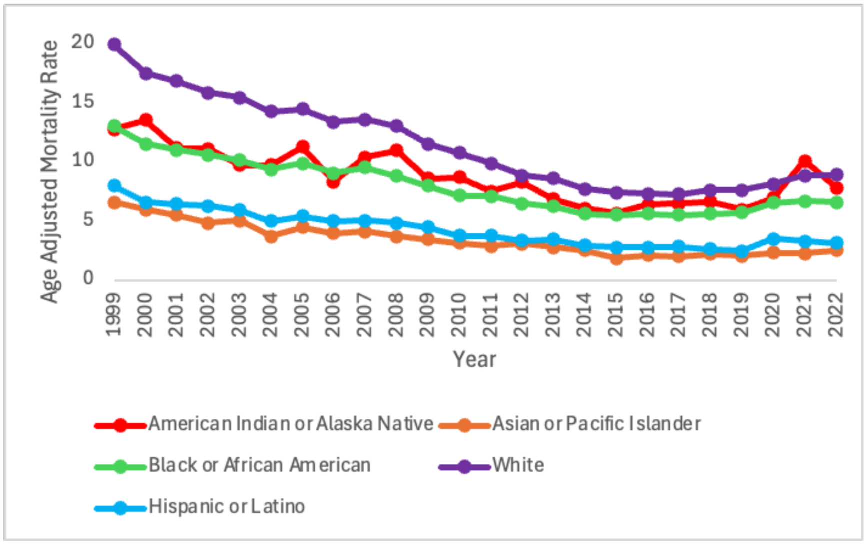

Supplemental Figure 2 - Emphysema Race Age Adjusted Mortality Rates

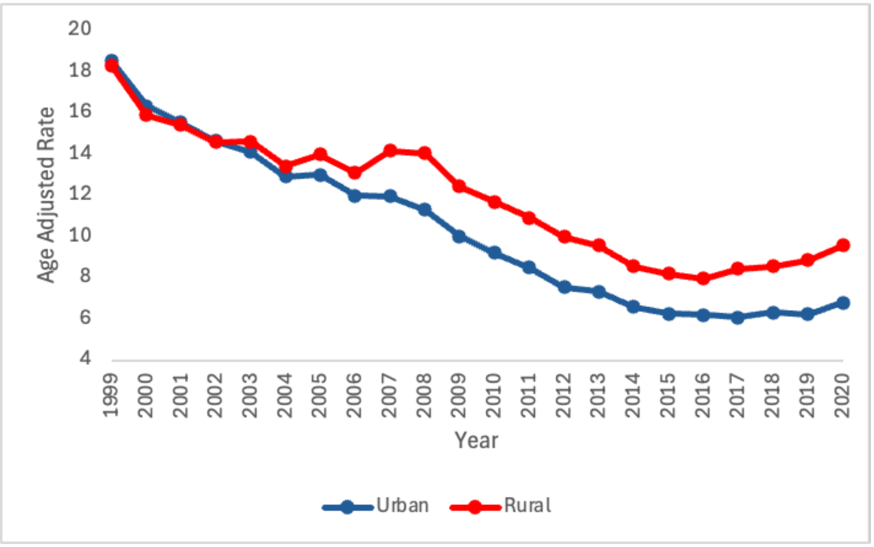

Supplemental Figure 3 - Emphysema Urban vs Rural Age Adjusted Mortality Rates

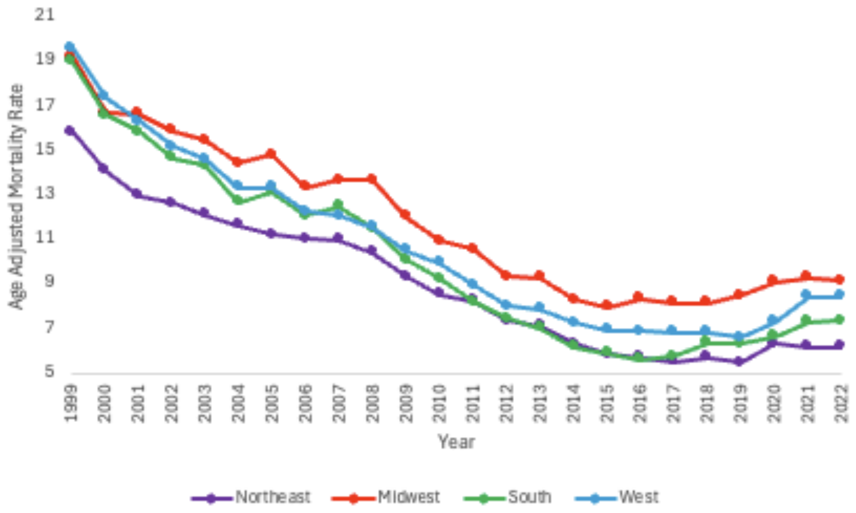

Supplemental Figure 4 - Emphysema Regional Age Adjusted Mortality Rates

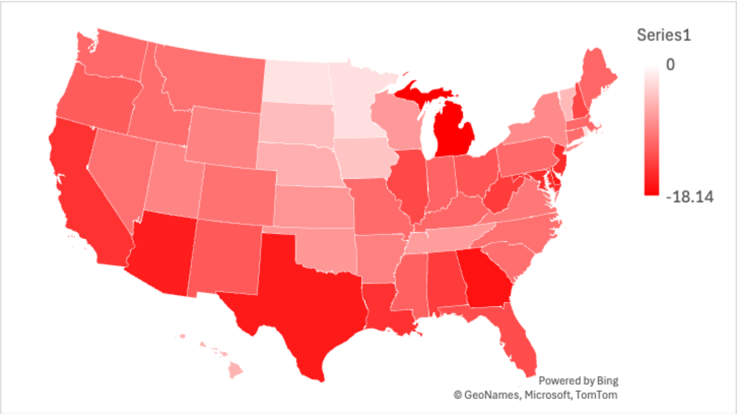

Supplemental Figure 5 - Emphysema State Age Adjusted Mortality Rates 1999-2019

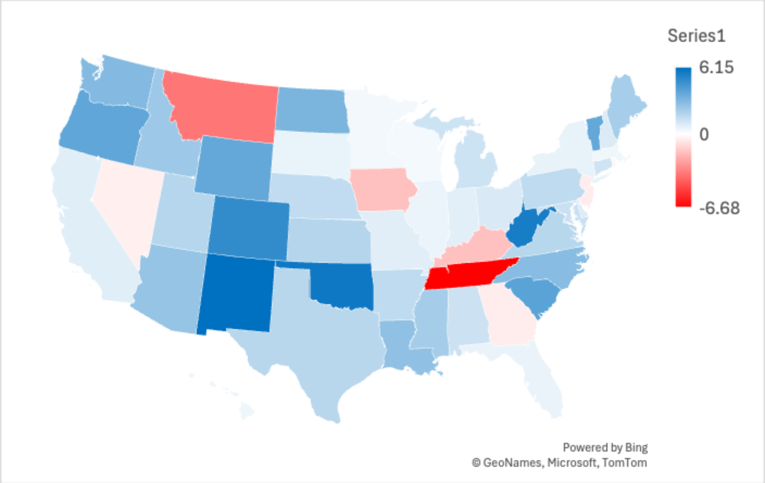

Supplemental Figure 6 - Emphysema State Age Adjusted Mortality Rates 2019-2022

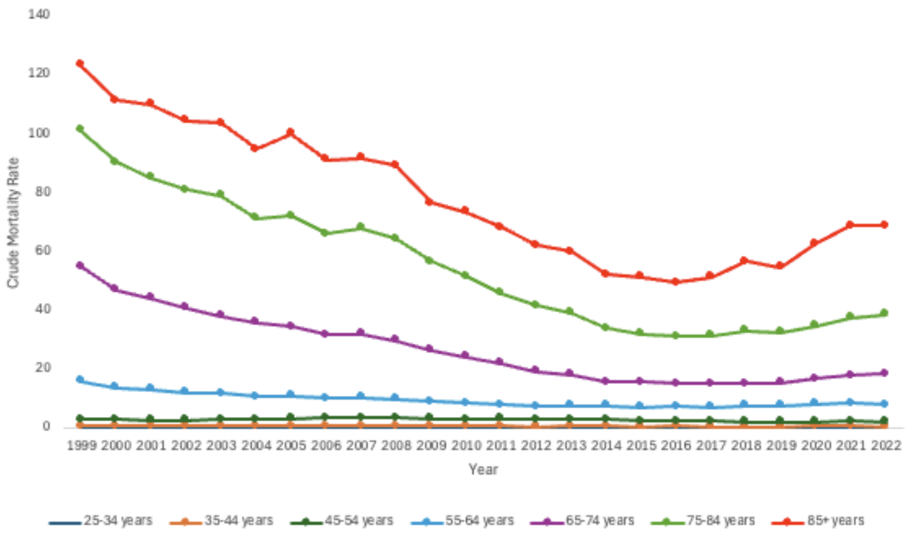

Supplemental Figure 7 - Emphysema Age Crude Mortality Rates
